# Large Language Model–Driven Knowledge Graph Construction in Sepsis Care Using Multicenter Clinical Databases: Development and Usability Study

**DOI:** 10.2196/65537

**Published:** 2025-03-27

**Authors:** Hao Yang, Jiaxi Li, Chi Zhang, Alejandro Pazos Sierra, Bairong Shen

**Affiliations:** 1 Department of Critical Care Medicine, Joint Laboratory of Artificial Intelligence for Critical Care Medicine Frontiers Science Center for Disease-related Molecular Network, Institutes for Systems Genetics Sichuan University, West China Hospital Chengdu China; 2 Information Center, Engineering Research Center of Medical Information Technology, Ministry of Education West China Hospital Sichuan University Chengdu China; 3 Department of Computer Science and Information Technologies, Iberian Society of Telehealth and Telemedicine University of A Coruña A Coruña Spain; 4 Department of Clinical Laboratory Medicine Jinniu Maternity and Child Health Hospital of Chengdu Chengdu China; 5 Department of Computer Science and Information Technologies, Iberian Society of Telehealth and Telemedicine Research Center for Information and Communications Technologies, Biomedical Research Institute of A Coruña University of A Coruña A Coruña Spain; 6 Department of Critical Care Medicine, Joint Laboratory of Artifcial Intelligence for Critical Care Medicine Frontiers Science Center for Disease-related Molecular Network, Institutes for Systems Genetics Sichuan University, West China Hospital Chengdu China

**Keywords:** sepsis, knowledge graph, large language models, prompt engineering, real-world, GPT-4.0

## Abstract

**Background:**

Sepsis is a complex, life-threatening condition characterized by significant heterogeneity and vast amounts of unstructured data, posing substantial challenges for traditional knowledge graph construction methods. The integration of large language models (LLMs) with real-world data offers a promising avenue to address these challenges and enhance the understanding and management of sepsis.

**Objective:**

This study aims to develop a comprehensive sepsis knowledge graph by leveraging the capabilities of LLMs, specifically GPT-4.0, in conjunction with multicenter clinical databases. The goal is to improve the understanding of sepsis and provide actionable insights for clinical decision-making. We also established a multicenter sepsis database (MSD) to support this effort.

**Methods:**

We collected clinical guidelines, public databases, and real-world data from 3 major hospitals in Western China, encompassing 10,544 patients diagnosed with sepsis. Using GPT-4.0, we used advanced prompt engineering techniques for entity recognition and relationship extraction, which facilitated the construction of a nuanced sepsis knowledge graph.

**Results:**

We established a sepsis database with 10,544 patient records, including 8497 from West China Hospital, 690 from Shangjin Hospital, and 357 from Tianfu Hospital. The sepsis knowledge graph comprises of 1894 nodes and 2021 distinct relationships, encompassing nine entity concepts (diseases, symptoms, biomarkers, imaging examinations, etc) and 8 semantic relationships (complications, recommended medications, laboratory tests, etc). GPT-4.0 demonstrated superior performance in entity recognition and relationship extraction, achieving an *F*_1_-score of 76.76 on a sepsis-specific dataset, outperforming other models such as Qwen2 (43.77) and Llama3 (48.39). On the CMeEE dataset, GPT-4.0 achieved an *F*_1_-score of 65.42 using few-shot learning, surpassing traditional models such as BERT-CRF (62.11) and Med-BERT (60.66). Building upon this, we compiled a comprehensive sepsis knowledge graph, comprising of 1894 nodes and 2021 distinct relationships.

**Conclusions:**

This study represents a pioneering effort in using LLMs, particularly GPT-4.0, to construct a comprehensive sepsis knowledge graph. The innovative application of prompt engineering, combined with the integration of multicenter real-world data, has significantly enhanced the efficiency and accuracy of knowledge graph construction. The resulting knowledge graph provides a robust framework for understanding sepsis, supporting clinical decision-making, and facilitating further research. The success of this approach underscores the potential of LLMs in medical research and sets a new benchmark for future studies in sepsis and other complex medical conditions.

## Introduction

Sepsis, a critical condition leading to septic shock and multiple organ dysfunction syndrome in patients, often arises from severe trauma, surgery, or infections [1]. Despite advancements in diagnostics, therapeutics, and patient monitoring, the incidence and mortality rates of sepsis remain alarmingly high, posing a global medical challenge. Annually, it affects over 49 million individuals worldwide, with approximately 11 million fatalities, and mortality rates fluctuate between 15% and 25% [2]. This highlights the necessity for comprehensive research into sepsis to enhance prevention and treatment methods. Recent studies have increasingly concentrated on understanding its pathogenesis [3], clinical presentations [4], and treatment approaches [5]. However, the complexity and variability of sepsis complicate the development of effective treatments and rehabilitation strategies. Furthermore, survivors often endure long-term effects like cognitive dysfunction [6], indicating a gap in our understanding of postsepsis syndrome.

Constructing a powerful database specifically for sepsis is paramount. This initiative will significantly strengthen support for clinical data mining and big data applications, enabling the aggregation and analysis of vast datasets. Such progress could lay the groundwork for pioneering research. Furthermore, developing a comprehensive knowledge graph [7] on sepsis is crucial, as it will serve as a bridge between the deep application of databases and a more profound understanding. By closely integrating the detailed data from the database with the associative analysis capabilities of the knowledge graph, we can more effectively unveil the complex mechanisms of sepsis, propelling deep research advancements in this field. In addition to the database, by integrating findings from related studies, clinical presentations, and treatment methods on sepsis, this knowledge graph could offer multidimensional and multilevel information. It aims to delve into the pathogenesis, clinical manifestations, and conventional treatment options for sepsis, providing a deeper insight into its complexities. This will serve as a scientific basis for formulating more effective preventive and treatment strategies, as well as providing clinicians with real-time and comprehensive reference materials to accurately assess patients’ conditions and optimize treatment plans.

However, the relationship extraction step [8] in knowledge graph construction is a critical juncture, determining the accuracy and completeness of the relationships between entities. Currently, deep learning models based on natural language processing technology are commonly used for clinical text entity recognition and relationship extraction. This process relies on professional data labeling, neural network model design, and large-sample model training, making it a time-consuming and labor-intensive task. Recently, large language models (LLMs) such as GPT-4.0 [9] and LLaMA [10] have revolutionized the field of natural language processing, offering new insights to address this issue. These models possess the ability to be widely applied to various downstream tasks, such as named entity recognition (NER) [11] and relationship extraction [12]. Meanwhile, through the design and optimization of prompt engineering [13], large models’ capability to handle complex task scenarios is enhanced and widely applied in the medical field [14]. Floridi and Chiriatti [15] evaluated multiple pretrained and fine-tuned large language models (LLMs) on their ability to extract adverse events from the Vaccine Adverse Event Reporting System (VAERS) notes. The fine-tuned adverse events LLM achieved an impressive average micro *F*_1_-score of 0.704, demonstrating the potential of LLMs in natural language processing tasks. Hu et al [11] assessed ChatGPT’s ability to perform zero-shot clinical NER as defined by the 2010 i2b2 challenge and compared it to models based on GPT-3 [15] and Bio-Clinical BERT (Bidirectional Encoder Representations from Transformers) [16]. The results revealed that while ChatGPT’s zero-shot performance was not as strong as fine-tuned BERT clinical models, its *F*_1_-score reached 0.628 under relaxed matching criteria, indicating reasonable performance. However, these studies also highlighted some limitations, such as errors from hallucinations (ie, generating inaccurate, meaningless, or contextually irrelevant outputs) [17] and insensitivity to negation words [18].

In this research, we aim to establish a multicenter sepsis database (MSD), providing a richer and more diverse dataset that is crucial for the in-depth analysis and understanding of sepsis. Using GPT4.0 for entity recognition and relation extraction, we aim to construct a comprehensive sepsis knowledge graph, using real-world databases supplemented by clinical guidelines and relevant public databases. This detailed knowledge graph is intended to be an exhaustive reference for clinicians, enhancing their comprehension and treatment approaches for sepsis. By the application of these advanced technologies, we strive to improve the construction quality and efficiency of the knowledge graph, providing strong support for sepsis research and clinical management.

## Methods

### Data Collection and Database Construction

**Figure 1:**
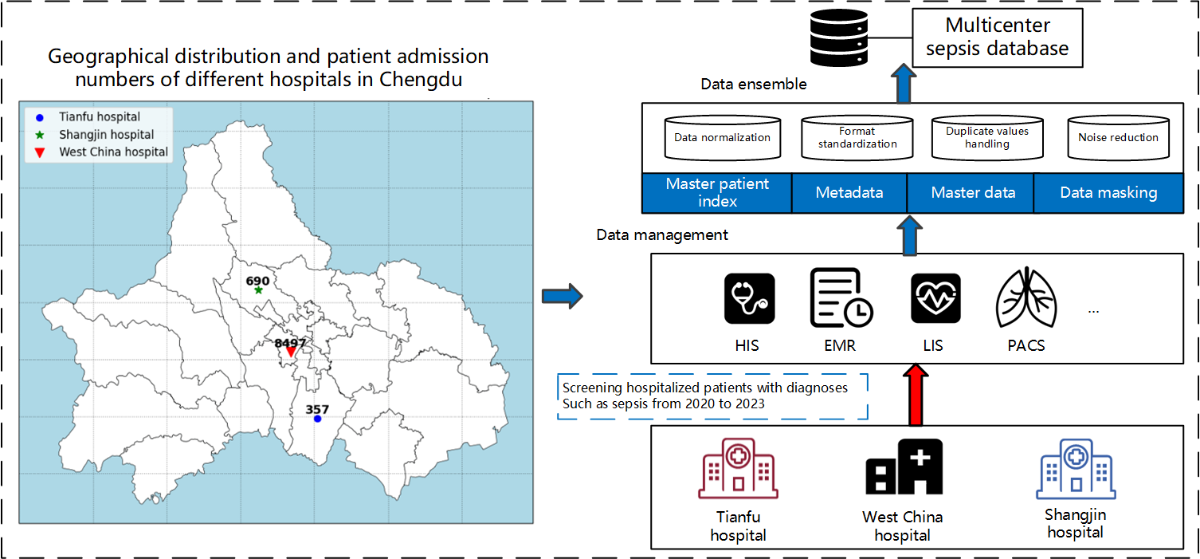
Overview of the sepsis dataset from West China Hospital of Sichuan University, Shangjin Hospital, and Tianfu Hospital (2020-2023).

The dataset originates from prominent tertiary hospitals in western China, including the West China Hospital of Sichuan University, Shangjin Hospital, and Tianfu Hospital. It consists of records from hospitalized patients diagnosed with sepsis between 2020 to 2023. The inclusion criteria for the dataset were patients whose discharge diagnoses prominently featured sepsis within the specified timeframe (2020-2023). For these patients, comprehensive data were collected, including baseline demographics, laboratory test results, and electronic medical records documenting admission and discharge details, among other pertinent information, as shown in Figure 1. The structure of the database is shown as an Entity Relationship Diagram (Figure S1, Multimedia Appendix 1). Before any EHR data is processed by the GPT-4 model, all personal identifiers, such as names, addresses, and patient IDs, are removed following standard deidentification protocols. This ensures that no identifiable information is included during the relationship extraction and entity recognition process.

### Sepsis Expert Consensus and Guidelines 

Incorporating data from expert consensus statements and clinical guidelines on sepsis establishes a foundational understanding of best practices and current recommendations in the field. These authoritative sources significantly contribute to the development of a comprehensive knowledge base. The primary data source is PubMed, and our search query is as follows:

(sepsis[ti] and guideline[pt] and 2020/01/01:2023/12/01[pdat]) or (sepsis[ti] and review[pt] and 2020/01/01:2023/12/01[pdat])

This search is designed to retrieve all expert guidelines and literature reviews on sepsis published in the last 3 years, serving as a vital knowledge repository for subsequent relationship extraction. To automate the process of retrieving articles from PubMed, we used the Biopython library [19] which provides an efficient way to interact with the PubMed database. In addition, we integrated biomarker data from MetaSepsisBase [16], an open database for sepsis and the first sepsis biomarker knowledge database ever constructed. The database comprises biomedical information on 320 sepsis biomarkers, with 450 records sourced from PubMed and annotated through the NCBI Gene database [17] and HGNC database [18]. This initiative provides a solid knowledge foundation for the construction of a sepsis knowledge graph.

### The Construction of Knowledge Graph

The construction process of the sepsis knowledge graph primarily uses an entity-based approach, involving key steps such as data preprocessing, analysis, entity construction [20], knowledge extraction [21], fusion [22], and storage. In contrast to traditional knowledge graph construction methods, this study leverages GPT-4.0 to extract knowledge and conduct initial data mining on raw data, enhancing the accuracy of the resulting knowledge graph.

### Building the Entity of Knowledge Graph

The primary objective of entity construction [23] is to acquire, describe, and represent knowledge within a specific domain, fostering a shared understanding of the sepsis field. This involves identifying widely accepted terms, providing precise definitions, and establishing their interrelationships across different levels of formal patterns. In our research, the entity definitions and their interrelationships are presented in Tables 1 and 2.

**Table 1:** Definitions of entity concepts in the sepsis knowledge graph based on multicenter clinical data (N=10,544) from 3 hospitals in Western China (2020-2023).

Number	Concept terms	Attributes	Meaning
1	Diseases	Canonical name, concept ID, TUIs^a^, definition, reference, and synonyms	Disease is an atypical life process that occurs due to disrupted self-regulation in the body under specific conditions after exposure to pathogenic damage.
2	Symptoms	Canonical name, concept ID, TUIs, duration, severity, frequency, and temporal pattern	Subjective atypical sensations or objective pathological changes in a patient resulting from a series of physiological, metabolic, and morphological abnormalities during the disease process.
3	Imaging examination	Canonical name, concept ID, TUIs, type, body region, and imaging findings	Imaging examination is a technique used to visualize the interior of a body for clinical analysis and medical intervention.
4	Biomarkers	Canonical name, concept ID, TUIs, biomarker type, reference, synonyms, cut-off value, sensitivity, and specificity	Measurable substances in the body used to indicate physiological status, the presence of disease, or disease progression.
5	Laboratory test	Canonical name, concept ID, TUIs, normal range, unit, and measured value	Physical or chemical examinations conducted in a laboratory to determine the content, nature, concentration, quantity, and other characteristics of submitted substances.
6	Subtypes	Canonical name, concept ID, TUIs, definition, reference, and synonyms	Subdivisions within a category or group based on specific features or characteristics. For example, disease subtypes may indicate different pathological features or symptom presentations.
7	Pathogenic mechanism	Canonical name, concept ID, UIs^b^, type, infection source, reference, biomarkers, and guidelines	Biological or biochemical processes leading to the occurrence of disease, including factors such as pathogen infection and genetic mutations.
8	Pharmacotherapy	Canonical name, concept ID, TUIs, medication name, drug class, dosage, duration of treatment, adverse effects, drug interactions, and formulations	Substances used for preventing, treating, and diagnosing diseases. In theory, drugs encompass chemical substances that can influence the physiological functions and cellular metabolic activities of the body’s organs.
9	Surgery	Canonical name, concept ID, TUIs, anesthesia type, guidelines, complications, recovery time, and level	A procedure involving the use of instruments, performed by a surgeon or other specialized personnel, to enter the human body or other biological tissues. It involves the application of external force to eliminate pathology, alter structures, or implant foreign materials.

^a^TUI: Type Unique Identifier, representing the semantic category of a concept in UMLS..

^b^UI: Unique Identifier, used to uniquely distinguish a concept or term in UMLS..

**Table 2:** Definitions of semantic relations in sepsis data based on multicenter clinical data (N=10,544) from 3 hospitals in Western China (2020-2023).

Number	Concept terms	Meaning
1	Complications	Disease development may give rise to another disease or the occurrence of additional symptoms.
2	Has symptom	Describing the correlation between diseases and symptoms.
3	Recommended imaging examination	Recommended imaging examination for patients with sepsis.
4	The related biomarkers	Biomarkers associated with sepsis.
5	Treat	Surgical treatment modalities associated with sepsis.
6	Recommended laboratory tests	Routine laboratory tests associated with sepsis.
7	Caused by	The underlying factors contributing to the onset of sepsis.
8	Recommended medication	Pharmacological interventions in the treatment process of sepsis.

**Figure 2:**
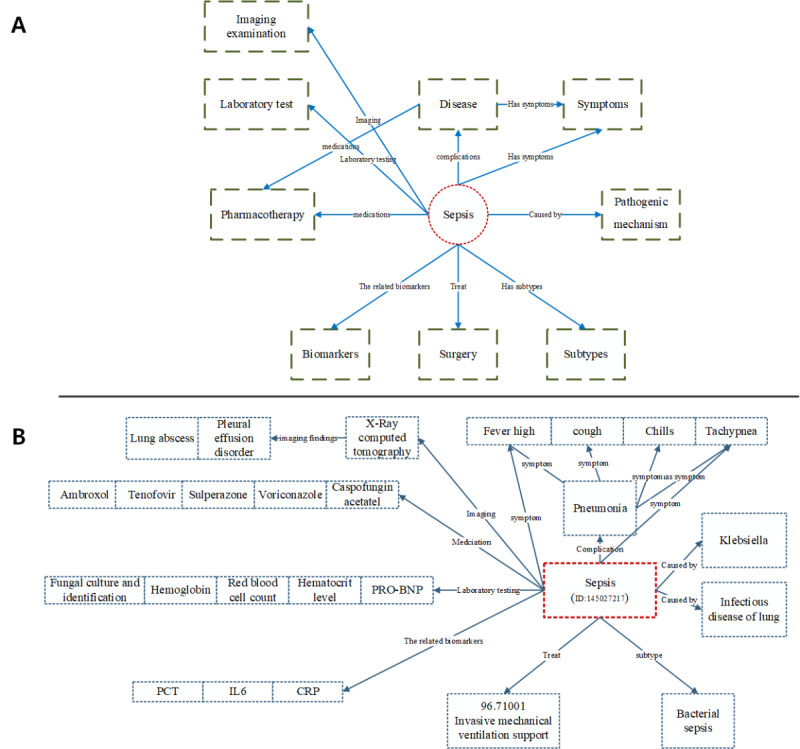
Construction of the sepsis knowledge graph using top-down and bottom-up approaches. (A) Pattern layer: in the top-down approach, a structured pattern layer is defined based on logical relationships and hierarchical structures. (B) Data Layer: the bottom-up approach extracts entities and attributes from diverse data sources. CRP: C-reactive protein; IL: interleukin; PCT: Procalcitonin; PRO-BNP: Pro-B-Type Natriuretic Peptide (Pro-Brain Natriuretic Peptide).

The construction methods of knowledge graph technology are typically categorized into two approaches: top-down and bottom-up [24]. The top-down approach involves defining a pattern layer based on logical relationships and hierarchical structures, followed by mapping data entities to this schema (as shown in Figure 2A). In contrast, the bottom-up approach focuses on extracting entities and attributes from diverse data sources for the data layer of the knowledge graph (as shown in Figure 2B). This process consolidates the extracted entities and attributes, optimizing the schema layer of the knowledge graph for iterative updates to the entity model. The top-down approach ensures that domain entities are enriched with professional knowledge and accuracy, while the bottom-up approach enhances their practicality. Given the unique characteristics of sepsis, our research adopts both approaches to construct the sepsis knowledge graph. This dual approach aims to improve the educational value, accuracy, and practical utility of the knowledge graph.

### Prompt Engineering and Model Evaluation

In this study, we used the approach of LLMs prompt engineering for knowledge extraction. By harnessing the powerful language understanding and pattern recognition capabilities of GPT-4.0, we guided the model to generate sepsis-specific prompts, extracting relevant information. The advantage of this approach lies in its ability to handle complex structures and contexts in natural language without manually specifying intricate rules or patterns.

Compared with traditional knowledge extraction methods, LLM-based approaches offer several advantages. First, LLMs can autonomously learn the grammar and semantic rules of language, making them more adaptable to the intricate expressions within this domain [24]. Second, due to pretraining on large-scale corpora, LLMs acquire rich background knowledge, enhancing their contextual awareness when understanding texts in a specific domain [25]. In addition, LLMs excel at handling ambiguous and unclear language expressions, improving the robustness of knowledge extraction [26].

**Figure 3:**
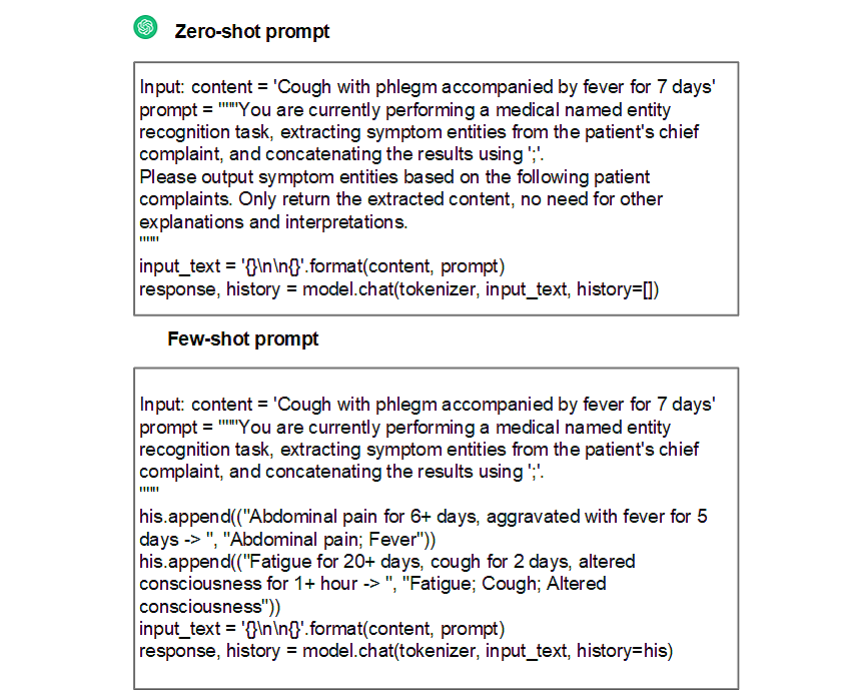
Prompt design for zero-shot and few-shot named entity recognition tasks in medical Chinese.

To enhance the effectiveness of our approach, we introduced zero-shot [27] and few-shot [28] learning techniques, as shown in Textbox 1. By leveraging few-shot learning within LLMs, we enable the model to quickly adapt to new sepsis-related texts even with limited examples, allowing the model to flexibly adjust to the specific context and linguistic expressions of a specialized domain. This adds flexibility and personalization to the knowledge extraction process, further improving the model’s performance and adaptability. To validate the effectiveness of prompt engineering with GPT-4.0, we selected the CMeEE (Chinese Medical Entity Extraction) public dataset [29] for the NER task (as shown in Figure 3), establishing a baseline performance by comparing it with traditional models such as Med-BERT [30] and LSTM (long short-term memory) [31]. This comparison demonstrates GPT-4.0’s adaptability and flexibility in processing complex medical texts, highlighting the potential of large language models in knowledge graph construction. In addition, to evaluate GPT-4.0’s recognition performance in our study, we created a dataset focused on sepsis. Due to the high cost of manual annotation, a medical expert labeled named entities in the electronic medical records (EMRs) of 61 randomly selected patients, with sample annotations provided in the Multimedia Appendix 1. We further assessed GPT-4.0’s performance in natural language processing tasks by comparing it with other large models (Qwen2 [Alibaba Cloud], Llama3 [Meta AI], and ChatGPT 3.5 [OpenAI]). The models were evaluated using the *F*_1_-score, which reflects overall performance by calculating true positives, false positives, and false negatives, summarizing both precision and recall. *F*_1_-score, the harmonic mean of precision and recall, provides a comprehensive measure of model effectiveness.

**Prompt design for sepsis relation extraction in zero-shot and few-shot learning scenarios.**

**Prompt design**

Suppose you are an entity-relationship triple extraction model. I'll give you a list of head entity types: subject types, list of tail entity types: object types, list of relations: relations. Give you a sentence, please extract the subject and object in the sentence based on these three lists and form a triplet in the form of (subject, relation, object).

relations: ['Clinical Presentation ', 'Surgical treatment', 'biomarker', 'subclass', 'pathogenesis', 'laboratory testing', 'Imaging examination', 'Complications', 'Medication']

The given sentence is \n {triple list}

Examples(few-shot)

examples = [

{

“text”: “In adults, about 1/3 of AHF (Acute Heart Failure) patients may develop concurrent fungal infections, primarily caused by Candida albicans. Circulatory system issues may lead to sinus bradycardia, with a slower heart rate occurring relatively late, and in a minority of cases, sudden cardiac arrest can occur.”,

“Triple list”: [

[“AHF”, “Complications”, “sinus bradycardia”], [“AHF”, “Complications”, “fungal infection”], [“Fungal infection”, “Biomarker”, “Candida albicans”]

]

},

{

“text”: “Pancreatic cancer, after 4 months of initial treatment, showed a pancreatic mass with liver metastasis in the upper abdominal ultrasound examination.”,

“Triple list”: [

[“Pancreatic cancer”, “Clinical Presentation”, “pancreatic mass”], [“Pancreatic cancer”, “Imaging examination”, “upper abdominal ultrasound examination”], [“Pancreatic cancer”, “Clinical Presentation”, “liver metastasis”]

]

}

]

### Knowledge Fusion

In our research, we used the entity-linker module from scispaCy [32], which incorporates the Unified Medical Language System (UMLS) [33] terminology standards to construct a comprehensive knowledge base. UMLS facilitated the alignment of terms, such as mapping “Hypertension” to the standardized concept “Hypertensive disease (C0020538).” To ensure effective text matching, we standardized and cleaned entity names across different data sources. This process included unifying letter case, removing punctuation, and handling abbreviations, allowing us to standardize terms such as “Heart Failure” and “HF” to a consistent terminology. Following this initial processing, we used text similarity algorithms for entity matching, using algorithms based on word embeddings and Jaccard similarity to calculate similarity scores between entity names. During the alignment process, we established a similarity threshold of 0.85, ensuring that only highly similar entities were recognized as corresponding. For entities with similarity values below this threshold, we retained their original values and used manual review to address any potential ambiguities, thereby ensuring the precision and credibility of the final alignment results. This meticulously designed workflow not only enhances the efficiency of entity alignment but also safeguards the accuracy and reliability of our research outcomes.

### Platform Implementation

**Figure 4:**
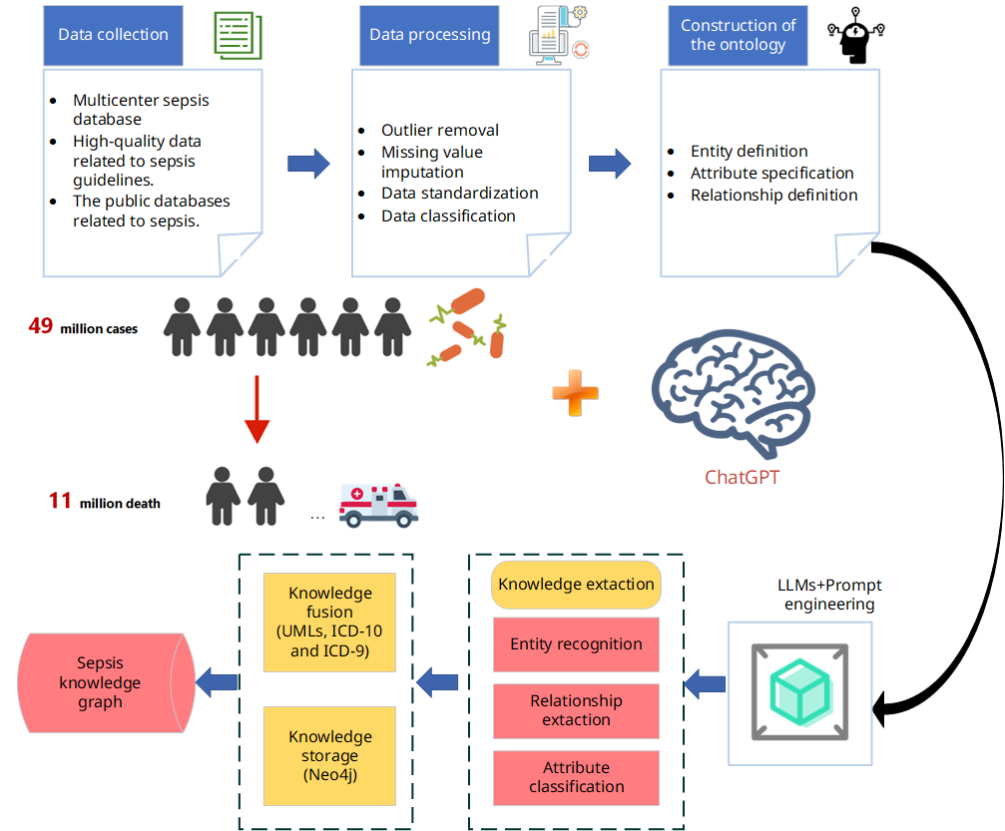
Framework of the multicenter sepsis knowledge graph construction study (n=10,544 patients with sepsis) integrating GPT-4 and Neo4j technologies. The flow diagram shows retrospective data collection from 3 tertiary hospitals in Western China (West China Hospital, Shangjin Hospital, and Tianfu Hospital; 2020-2023), multimodal data processing through LLM-driven entity recognition, and graph database implementation. LLM: large language model; MSD: multicenter sepsis database.

MSD performed data management using MySQL software (version 5.7; Oracle Corp). The web pages were developed with Bootstrap 4.0 (The Bootstrap Team) and used the Flask framework. Several JavaScript plugins, such as Datatable (version 1.10.10; SpryMedia Ltd) and ECharts (version 5.0; Baidu), were applied for creating data tables and visualizations. We used the Neo4j graph database [34] for knowledge storage. The underlying storage principles of this graph database involve the use of nodes, edges, and properties to store graph data. Neo4j is currently recognized as one of the most popular high-performance NoSQL graph databases [35], known for its high availability, stability, scalability, and robust intuitiveness. In this research, open-source Python libraries (such as py2neo [36] were used in conjunction with OpenAI interfaces to accomplish knowledge extraction and to add or modify relationships and nodes. Specifically, we used the GPT-4-turbo model accessed via the application programming interface for our artificial intelligence model, and for Scispacy, we used the en_core_sci_sm model. The overall research and construction process is shown in Figure 4.

### Ethical Considerations

This study was approved by the Medical Ethics Committee of West China Hospital, Sichuan University (approval number 2024-126). The research involved a secondary analysis of deidentified patient data, which was conducted in accordance with the original informed consent obtained during data collection, allowing for subsequent analyses without requiring additional consent. To ensure participant privacy and confidentiality, all personally identifiable information was removed before analysis. No financial compensation was provided to participants for the use of their data in this study. Furthermore, this manuscript does not contain any images that could potentially identify individuals. The relevant consent forms have been submitted as part of the supplementary materials.

## Results

In this study, we established an MSD [37] using real-world data from 3 major hospitals in Western China. The database spans the period from 2020 to 2023, covering a total of 10,544 patients diagnosed with sepsis. Each hospital contributed a significant number of patient records, with 8497 records from West China Hospital, 690 from Shangjin Hospital, and 357 from Tianfu Hospital. The MSD includes comprehensive data points such as baseline demographics, laboratory test results, EMRs, and detailed admission and discharge notes. This large-scale, multicenter sepsis dataset provides a solid foundation for constructing a highly detailed and clinically relevant sepsis knowledge graph.

Furthermore, using GPT-4.0, we conducted entity recognition and relationship extraction based on the MSD, expert consensus, and open data sources. Subsequently, we achieved knowledge integration by benchmarking against the UMLS. As a result, we present the definitions of key entities and their semantic relationships in Tables 1 and 2. Table 1 outlines key entities, such as diseases, symptoms, biomarkers, and imaging examinations, which are crucial for understanding the clinical characteristics of sepsis. For instance, the “Symptoms” entity captures the subjective and objective signs experienced by patients, while “Biomarkers” represent measurable substances that indicate the presence or progression of disease. The relationships between these entities, as defined in Table 2, provide insights into the complex dynamics of sepsis. Relationships such as “Has symptom” and “Complications” show how specific symptoms correlate with diseases and how disease progression may lead to additional health issues. We successfully constructed a comprehensive knowledge graph for sepsis, consisting of 1894 nodes and 2021 relationships. By integrating the definitions of entities and their interrelationships, our study aims to enhance the understanding of sepsis and supports informed clinical decision-making, ultimately contributing to improved patient outcomes. In addition, we have established relevant examples of sepsis ontology, as shown in Figure S7 in Multimedia Appendix 1.

Then, we introduced zero-shot and few-shot methods to explore the model’s performance in handling unseen or infrequent entities based on CMeEE, comparing them with conventional models such as BERT-CRF [34], BERT-wwm [35], Lattice-LSTM [36], and Med-BERT [30]. The results indicate that the few-shot approach using GPT-4 outperformed traditional models, achieving an *F*_1_-score of 65.42, which demonstrates its effectiveness in adapting to new entity types with limited annotated data. In addition, we constructed a Sepsis dataset, which consists of clinical records annotated by a medical expert, to further evaluate the models in a real-world health care context. Comparing performance across models, GPT-4 achieved a notable *F*_1_-score of 76.76, significantly surpassing other LLMs such as Qwen2 and Llama3, which indicates its strong capability in recognizing critical medical entities in this specialized domain. Tables 3 and 4 present the performance of different models on the CMeEE dataset and the Sepsis dataset.

**Table 3:** Zero-shot and few-shot learning performance comparison of GPT-4 versus BERT and Lattice-LSTM models on Chinese medical entity extraction dataset.

Model	CMeEE^a^		
	Precision	Recall	*F*_1_-score
BERT^b^-base	63.08	64.08	62.11
BERT-wwm	61.5	61.29	61.72
Lattice-LSTM^c^	46.34	43.60	49.44
Med-BERT	53.33	47.58	60.66
GPT-4 and Zero shot	64.07	68.97	59.82
GPT-4 and Few shot	65.31	64.89	65.73

^a^CMeEE: Chinese Medical Entity Extraction.

^b^BERT: Bidirectional Encoder Representations from Transformers.

^c^LSTM: long short-term memory.

**Table 4:** Performance comparison of GPT-4, Qwen2-72B, Llama3-70B, and GPT-3.5 models on the sepsis dataset.

Model	Sepsis dataset		
	Precision	Recall	*F*_1_-score
Qwen2-72B	44.73	42.85	43.77
Llama3-70B	49.40	47.43	48.39
GPT-3.5	56.63	54.48	55.53
GPT-4 and Zero shot	72.12	70.48	71.29
GPT-4 and Few shot	77.73	75.81	76.76

### Major Complication of Sepsis

**Figure 5:**
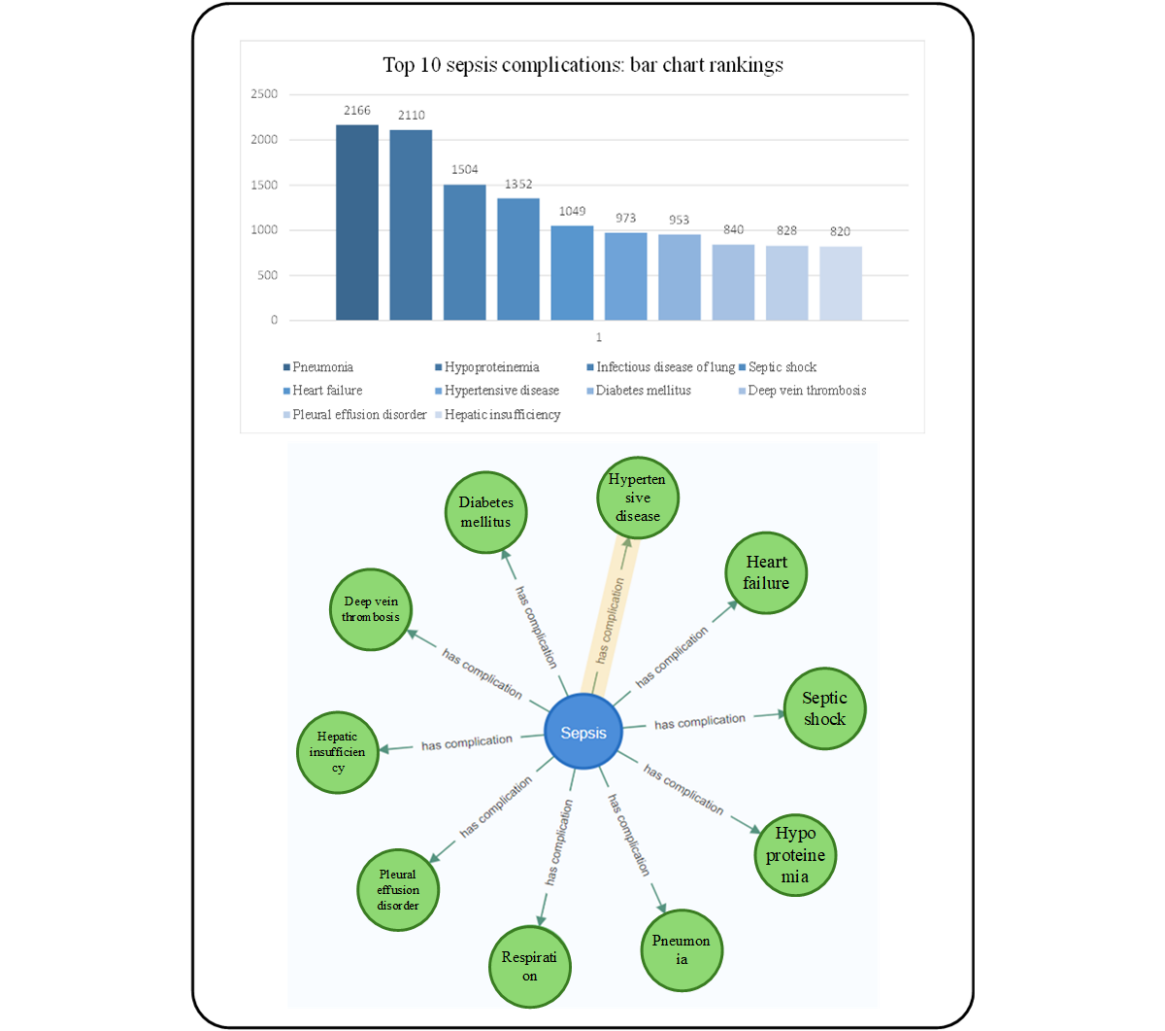
Top 10 sepsis complications identified through weighted relationship analysis in the multicenter sepsis database: Bar graph displays the prevalence of pneumonia (32.1%), hypoproteinemia (28.5%), septic shock (25.9%), and so on.

The investigation of complications associated with sepsis holds paramount significance in clinical research. In our study, we identified 203 complications-related nodes from the collected cases and literature (Figure 5). We queried the relationship between sepsis and complications, and through the query MATCH ()-[r:`has complication`]->() RETURN r ORDER BY r.weight DESC LIMIT 10.

### Sepsis Medication Statistics

**Figure 6:**
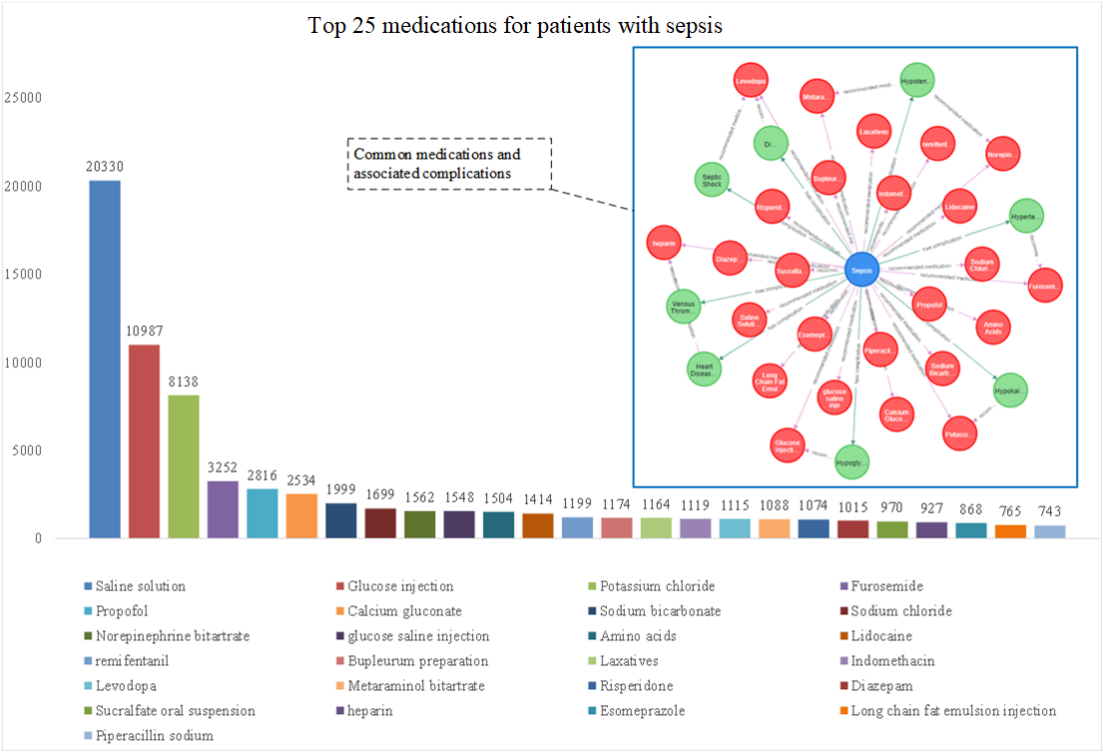
Pharmacological intervention patterns in sepsis management: a chord diagram depicting medication-complication relationships (physiological saline, glucose infusion, etc).

Sepsis is a severe systemic inflammatory response frequently triggered by infection. In the knowledge graph, entering the following code reveals the relationships between the most commonly used medications for sepsis and their associated complications (Figure 6). The following query was used: MATCH p=(n:medication )-[r:`recommended medication`]-() RETURN p order by r.weight ASC limit 25.

### Sepsis Symptom Statistics

**Figure 7:**
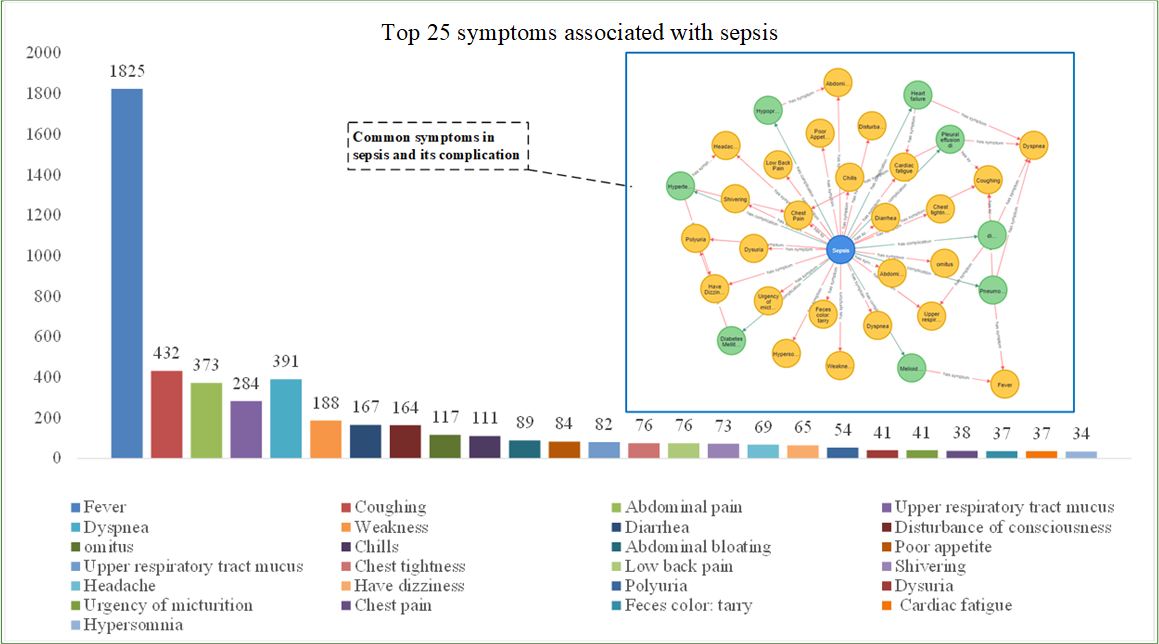
Knowledge graph representation of common symptoms of sepsis and their top 10 frequency.

Sepsis is defined as a comprehensive systemic inflammatory response, and its multifaceted nature is revealed through the statistical analysis of the top 25 symptoms observed in patients (Figure 7). Relevant queries were conducted in the knowledge graph, and the corresponding code is MATCH p=()-[r:`has symptom`]->() RETURN p order by r.weight asc.

### Clinical Applications

**Figure 8:**
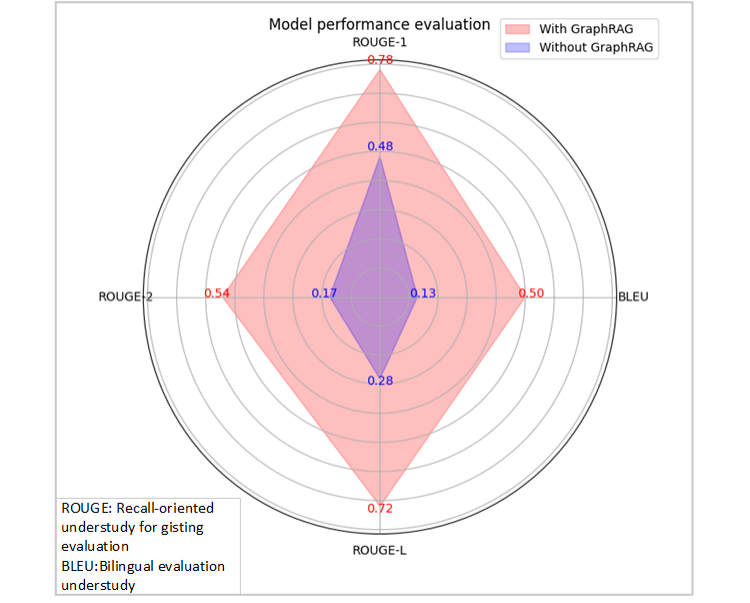
Comparative performance analysis of GraphRAG technology before and after implementation in sepsis clinical decision support.

Based on the results from the sepsis knowledge graph, we used GraphRAG [38] technology to assist large models in generating more precise decision support for clinicians. In comparison with traditional Retrieval Augmented Generation (RAG) technology, GraphRAG excels in handling complex relationships and multistep reasoning, providing more comprehensive and accurate answers, which is particularly crucial for the diagnosis and treatment of complex diseases like sepsis, as clinical decisions frequently involve multiple variables and potential interactions. Furthermore, GraphRAG enhances efficiency in information retrieval and customized summary generation, enabling clinicians to quickly access relevant information and recommendations related to patient conditions. This improvement in efficiency significantly bolsters the accuracy of clinical decisions, allowing physicians to make more informed judgments in complex clinical environments. With advancements in computational power, the integration of the sepsis knowledge graph and large models can consolidate data from electronic health records, laboratory tests, imaging, and critical care, enabling real-time analysis of patients’ clinical features and scoring systems (such as SOFA and APACHE II) to assist doctors in quickly identifying patients with sepsis across various risk levels and facilitating early warning systems. In the clinical decision-making process, the combination of the knowledge graph, RAG, and prompt engineering strategies offers treatment recommendations grounded in high-quality evidence, thereby enhancing the scientific and effective nature of decision-making. Currently, our clinical team has provided relevant case studies, showcased in supplementary materials, which include a comparison of GraphRAG technology before and after its implementation based on GPT-4. By using clinical gold standards and using BLEU [39], ROUGE-1, ROUGE-2, and ROUGE-L metrics [40], we compared the model's generated results, with BLEU primarily assessing the overlap of vocabulary between the generated text and reference text, reflecting the accuracy of the generated content, while ROUGE focuses on recall, emphasizing the comprehensiveness of the generated text in capturing reference content. Through a comprehensive analysis of these metrics, as shown in Figure 8, we further validated the effectiveness and advantages of GraphRAG in practical applications.

## Discussion

### Principal Findings

This study aimed to develop a framework for constructing a sepsis knowledge graph using large LLMs, leveraging real-world, multicenter clinical data. The core objective was to explore the feasibility and effectiveness of using LLM technologies, particularly GPT-4.0, to construct a comprehensive, domain-specific knowledge graph, focused on sepsis. Our findings indicate that GPT-4.0, through careful prompt engineering, is capable of accurately extracting entities and relationships from clinical data, outperforming traditional models such as BERT and LSTM. In particular, GPT-4.0 demonstrated the best few-shot performance on the CMeEE dataset, achieving an *F*_1_-score of 65.42, and outperformed other open-source models on the Sepsis dataset with an *F*_1_-score of 76.76. These results underscore the potential of LLMs for building specialized knowledge graphs in fields with limited data annotations, highlighting the advantages of the adaptability of LLMs across tasks and domains without the need for extensive model retraining.

The successful construction of a sepsis-specific knowledge graph marks a significant advancement in both knowledge extraction and clinical decision support. By using GPT-4.0’s pretrained capabilities, we were able to generate meaningful insights from small datasets, which typically require substantial domain-specific training. These findings align with other studies that have used large models such as GPT-4.0, GPT-3.5, LlaMA [10], and SOLAR [41], for biomedical relationship extraction. In particular, GPT-4.0 has shown superior performance in extracting biomedical relationships from semistructured data, achieving *F*_1_-scores above 0.881 [42], which further confirms its utility for creating knowledge graphs in health care. Our results also demonstrate that GPT-4.0 excels at quickly adapting to specialized domains, such as sepsis, and constructing medically relevant knowledge graphs without the extensive preprocessing and data annotation typically required in traditional machine learning methods. To contextualize these technical advancements within clinical applications, we conducted comparative analyses against established biomedical knowledge infrastructures, including BIOS [17], PrimeKG [7], and UMLS. This benchmarking reveals several critical advantages of our sepsis-focused approach. While BIOS prioritizes comprehensive ontology coverage through generalized semantic relationships [43], our model achieves superior clinical relevance through context-aware embeddings that preserve disease-specific pathophysiological hierarchies. In addition, while PrimeKG advances precision medicine via molecular-level drug-target interactions, our framework integrates multimodal clinical data streams, including 178 sepsis-associated biomarkers with temporal granularity, enabling dynamic risk stratification unavailable in existing resources. Furthermore, through systematic alignment with UMLS terminological standards, our implementation ensures interoperability without compromising domain specificity, addressing a critical limitation of conventional clinical knowledge graphs, which either sacrifice granularity for standardization or vice versa.

However, the sepsis knowledge graph we have constructed provides not only valuable insights into clinical practices but also enhances understanding of the complex pathophysiology of sepsis. The graph includes key complications such as pneumonia, hypoproteinemia, septic shock, and heart failure, as illustrated in Figure 5. By capturing these critical components, the graph aids in risk stratification and prognostication, ultimately improving treatment efficiency. In addition, our knowledge graph extends to therapeutic optimization. As shown in Figure 6, the system quantifies dynamic relationships between physiological saline administration, glucose metabolism correction, and electrolyte rebalancing interventions [41-43]. Machine learning modules further enable predictive modeling of antimicrobial efficacy based on pathogen susceptibility patterns and pharmacokinetic parameters [44,45], and simultaneously tracking analgesia requirements through nociception biomarkers [46]. This integrative approach allows for the real-time adjustment of therapeutic regimens in response to evolving inflammatory markers and organ dysfunction indicators. Our visualization platform also incorporates comprehensive symptom analytics, as shown in Figure 7. The graph’s temporal mapping functionality reveals progression trajectories from initial respiratory manifestations (eg, tachypnea, bronchial hypersecretion [47]) to subsequent multiorgan involvement, including gastrointestinal dysmotility [48], cardiovascular compromise [49], urinary symptoms [50], and neurological deterioration [51]. By correlating symptom clusters with biomarker profiles and treatment responses, the system enables early detection of sepsis-induced organ failure and provides decision support for personalized intervention strategies. This systematic integration of multimodal clinical data ultimately fosters a precision medicine paradigm in sepsis management, bridging pathophysiological insights with optimized therapeutic execution.

While the results are promising, we acknowledge several limitations in this study. Although GPT-4.0 demonstrated impressive entity recognition and relationship extraction capabilities, it occasionally produced erroneous outputs. For instance, the model recommended irrelevant laboratory tests (eg, “Blood cholesterol test”) for sepsis and misattributed causative factors, such as “Sepsis caused by exercise.” In addition, GPT-4.0 struggled with the nuanced semantics of traditional Chinese medicine terms, such as “Yin-Yang imbalance” and “Qi Deficiency,” leading to misinterpretations. These errors are significant as they could affect downstream clinical applications, such as diagnostic accuracy and therapeutic decision-making. However, the inherent limitations of GPT-4.0 in handling specialized medical terminologies and subtle relationships underscore the importance of a robust manual review process to ensure the accuracy of the knowledge graph. We have implemented such a review process to mitigate errors and maintain data integrity; however, further refinement of the model is necessary to reduce reliance on human intervention. In addition to these technical limitations, the use of GPT-4.0 in health care also raises important privacy and security concerns [52]. As noted, our study implemented stringent measures to protect patient data, including the elimination of personally identifiable information and adherence to relevant privacy regulations. Despite these precautions, the use of commercial models like GPT-4.0 poses risks related to data leakage and the potential exposure of sensitive patient information [53].

In future research, to ensure data security, we plan to deploy localized models, such as Llama 3.1 and Qwen 2, in secure environments, ensuring that all data processing activities are contained within controlled systems. By doing so, we aim to enhance data privacy and reduce the risk of data breaches, ensuring that the integrity of the knowledge graph remains intact while safeguarding patient confidentiality. Furthermore, we plan to further optimize the model’s accuracy and contextual understanding by incorporating advanced models such as Gemini [54] and Claude [55], which may provide more precise and contextually accurate results. We also aim to expand our dataset to include additional clinical and traditional Chinese medicine-related data to further enhance the robustness and adaptability of the knowledge graph. Fine-tuning techniques, such as Low-rank adaptation [56] and p-tuning [57], will be explored to improve both model precision and efficiency, ensuring that the knowledge graph captures a broader range of medical nuances. Furthermore, we will experiment with alternative prompt designs, including converting entity recognition tasks into classification tasks [58] or referencing the Teler taxonomy [59], which could improve the model’s ability to handle complex medical relationships more effectively. As part of this effort, we will also generate more gold standard data to compare the impact of various prompt strategies on knowledge graph accuracy.

In summary, this study highlights the potential of LLM-driven approaches, particularly GPT-4.0, in constructing accurate and effective medical knowledge graphs, even in data-scarce environments. The sepsis knowledge graph we developed not only enhances the understanding of sepsis but also illustrates the broader applicability of LLM-driven methods in creating domain-specific knowledge graphs across various medical fields. As large models like GPT-4.0 continue to evolve, their ability to process and interpret complex medical data will revolutionize how health care professionals access, interpret, and apply medical knowledge. The scalability and adaptability of this approach indicate its potential for widespread use in a variety of diseases, laying the foundation for personalized and precision medicine in clinical practice. In addition, the interactive and visual nature of the knowledge graph increases its practical utility, providing clinicians with a dynamic tool for both research and everyday decision-making.

### Conclusions

The successful establishment of the MSD, leveraging real-world data and the innovative application of LLMs like GPT-4.0, represents a significant leap forward in the understanding and management of sepsis. The use of prompt engineering to build a comprehensive sepsis knowledge graph not only showcases the potential of LLM technologies in medical research but also highlights their capacity for significant generalization and adaptation. Our findings, especially the enhanced performance of the few-shot model in entity identification and relationship understanding, underscore the efficiency and cost-effectiveness of using LLMs in the rapid development of specialized databases and knowledge graphs. This pioneering approach sets a new benchmark for future research and database development in the field of sepsis and potentially other medical domains.

## Data Availability

To ensure data security, the database currently does not support direct downloading on the website. Researchers in need can contact Professor Bairong Shen, head of the Disease Systems Genetics Research Institute at West China Hospital, by email at bairong.shen@scu.edu.cn

## Appendix

Multimedia Appendix 1 Additional information.

## Abbreviations

BERT: Bidirectional Encoder Representations from Transformers
CMeEE: Chinese Medical Entity Extraction
EMR: electronic medical record
LLM: large language model
LSTM: long short-term memory
MSD: multicenter sepsis database
NER: named entity recognition
RAG: Retrieval Augmented Generation
UMLS: Unified Medical Language System
VAERS: Vaccine Adverse Event Reporting System
