# Supplementary material for: Large Language Model–Driven Knowledge Graph Construction in Sepsis Care Using Multicenter Clinical Databases: Development and Usability Study
Source: J Med Internet Res. 2025 Mar 27;27:e65537. doi: 10.2196/65537 (PMC11986385; doi:10.2196/65537)
Supplement: Multimedia Appendix 1 [file jmir_v27i1e65537_app1.docx]

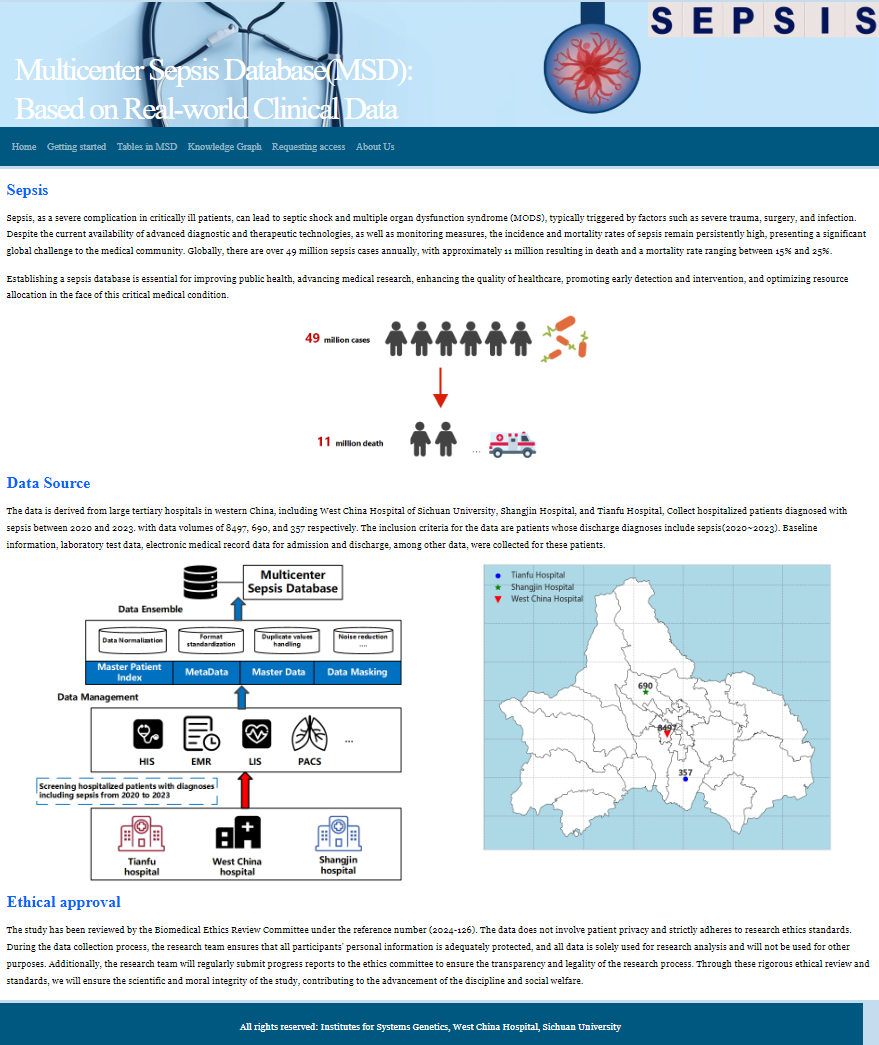


Supplementary Figure S1. The web page of Multicenter Sepsis Database (MSD)


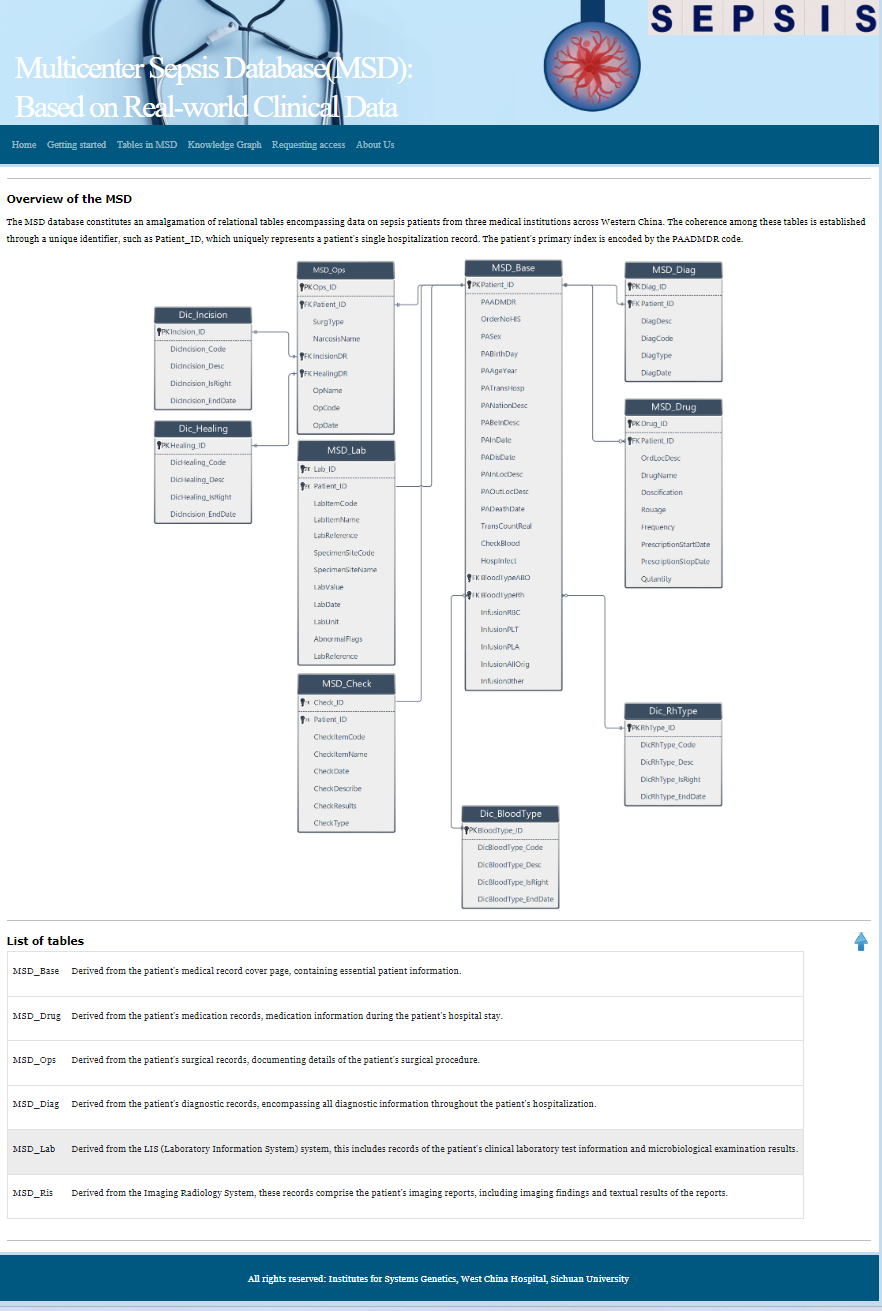


Supplementary Figure S2. The web page of Multicenter Sepsis Database (overview of the MSD)


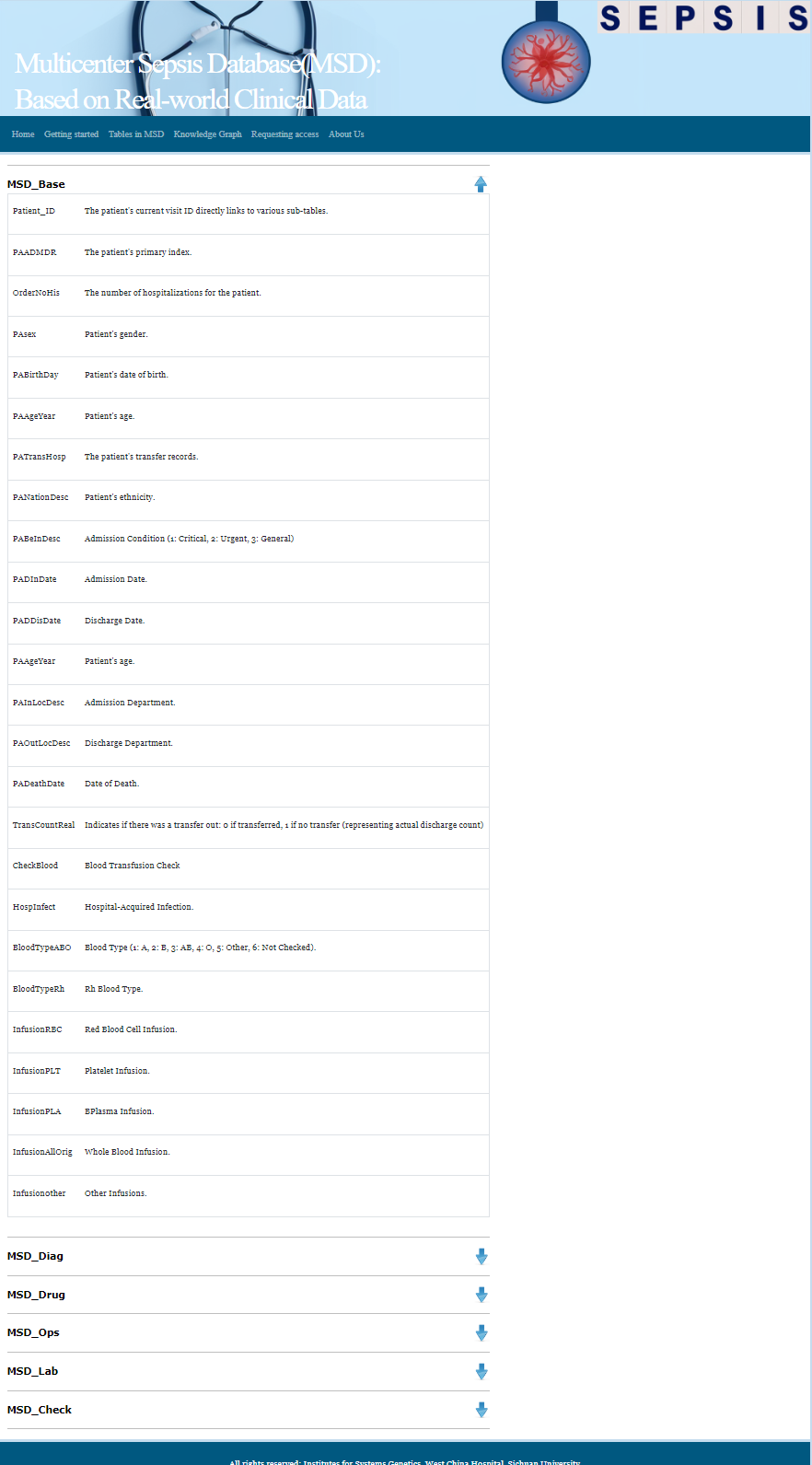


Supplementary Figure S3. Data Table Structure


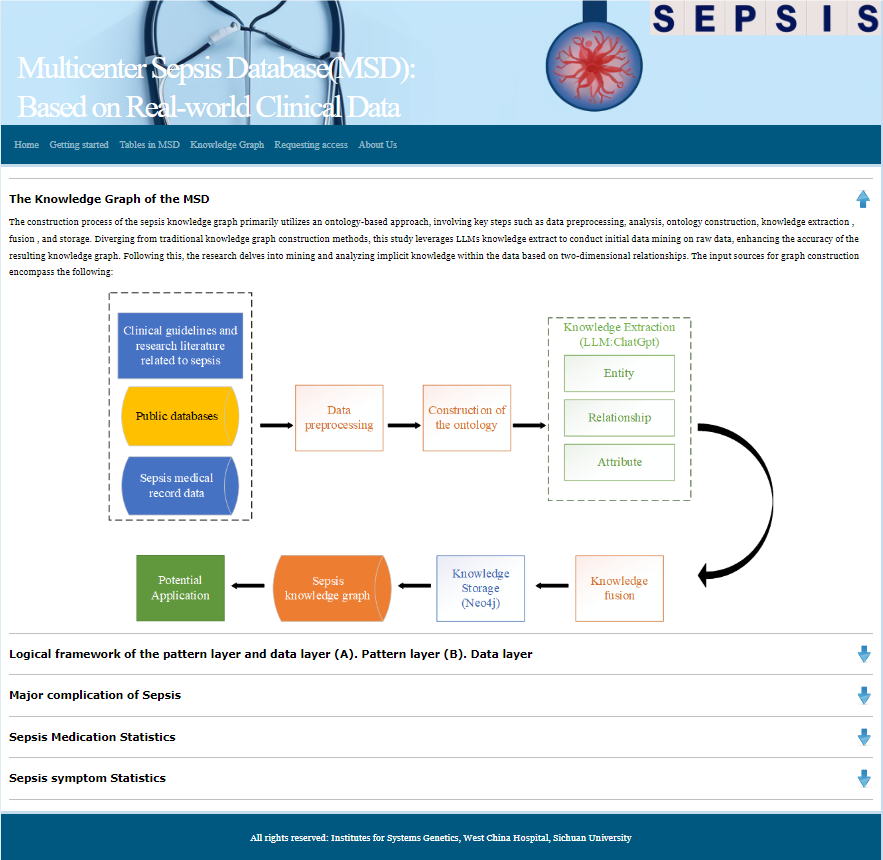


Supplementary Figure S4. The knowledge graph of the MSD


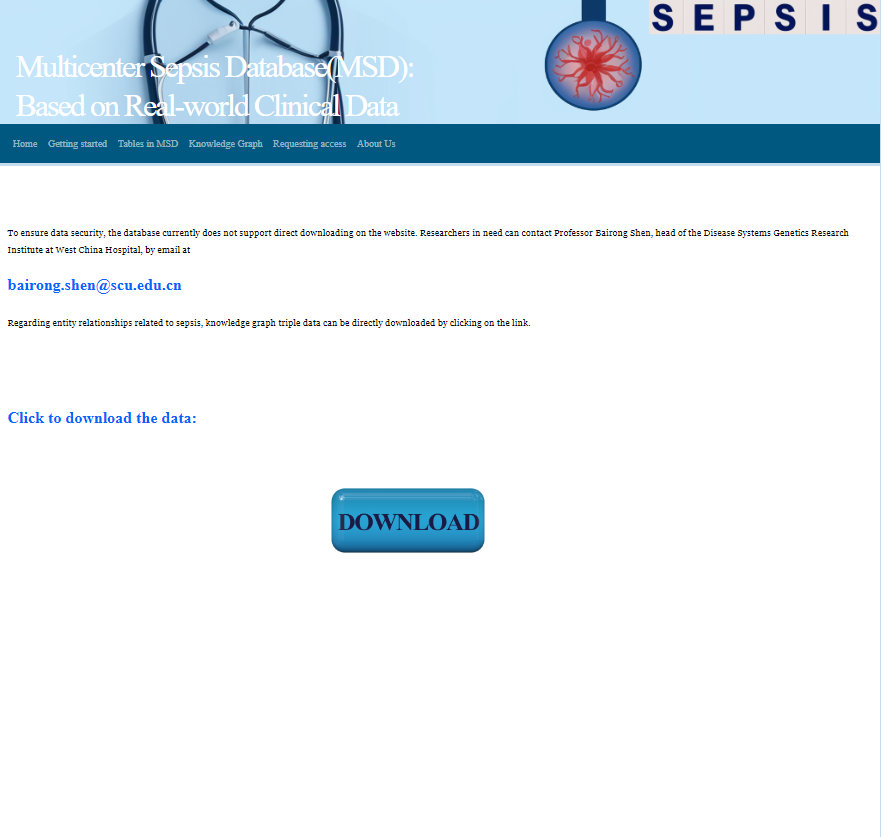


Supplementary Figure S5. The download page


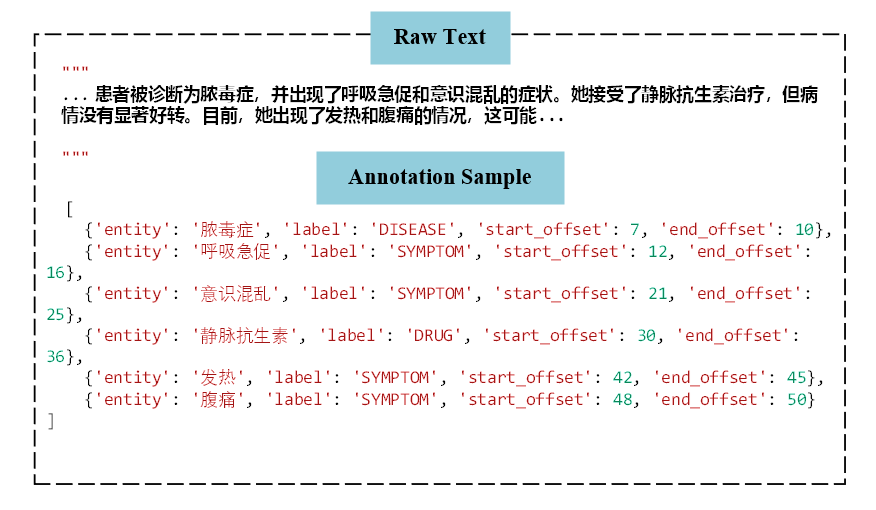


Supplementary Figure S6. The annotation sample


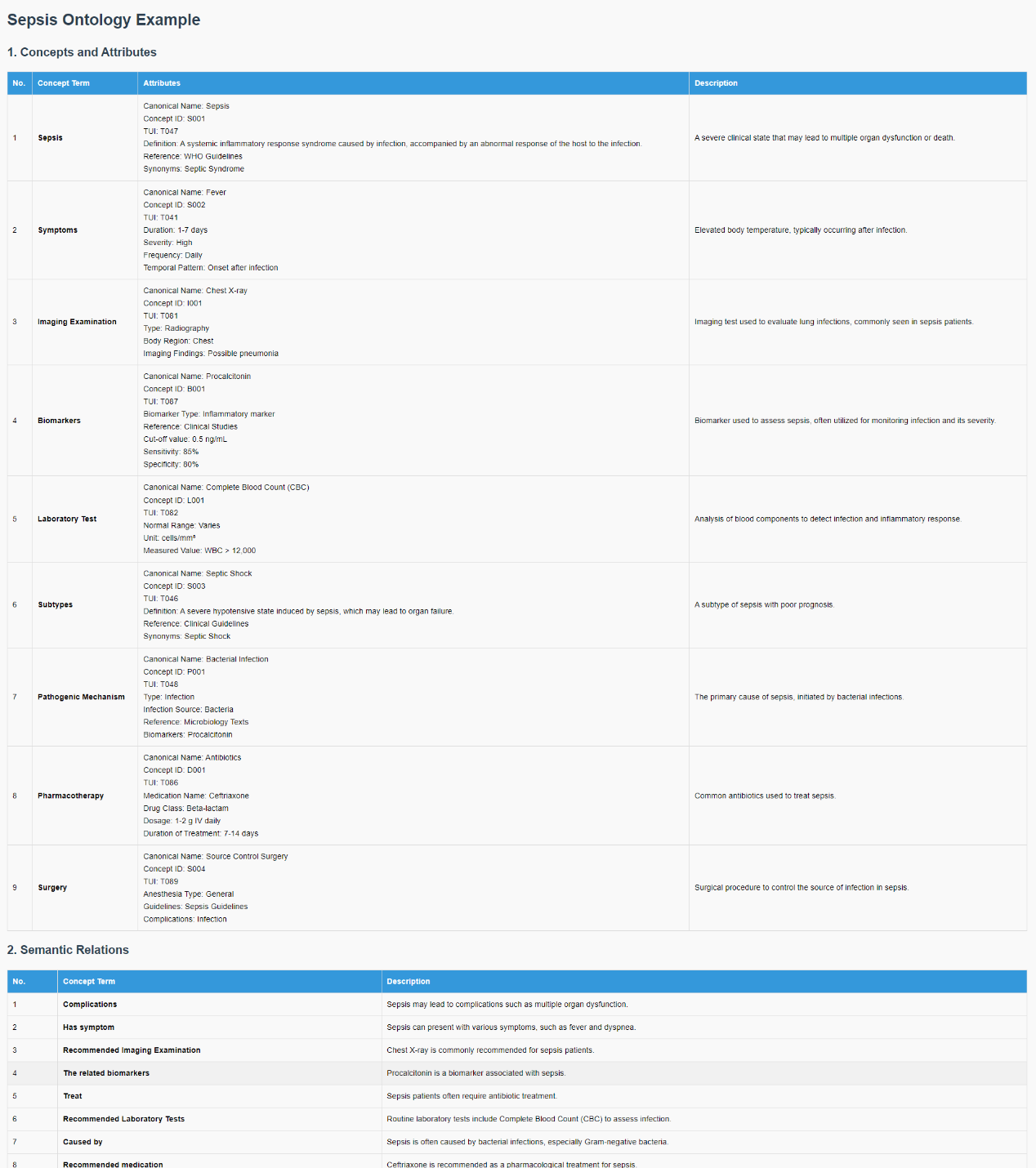


Supplementary Figure S7. The sepsis ontology example


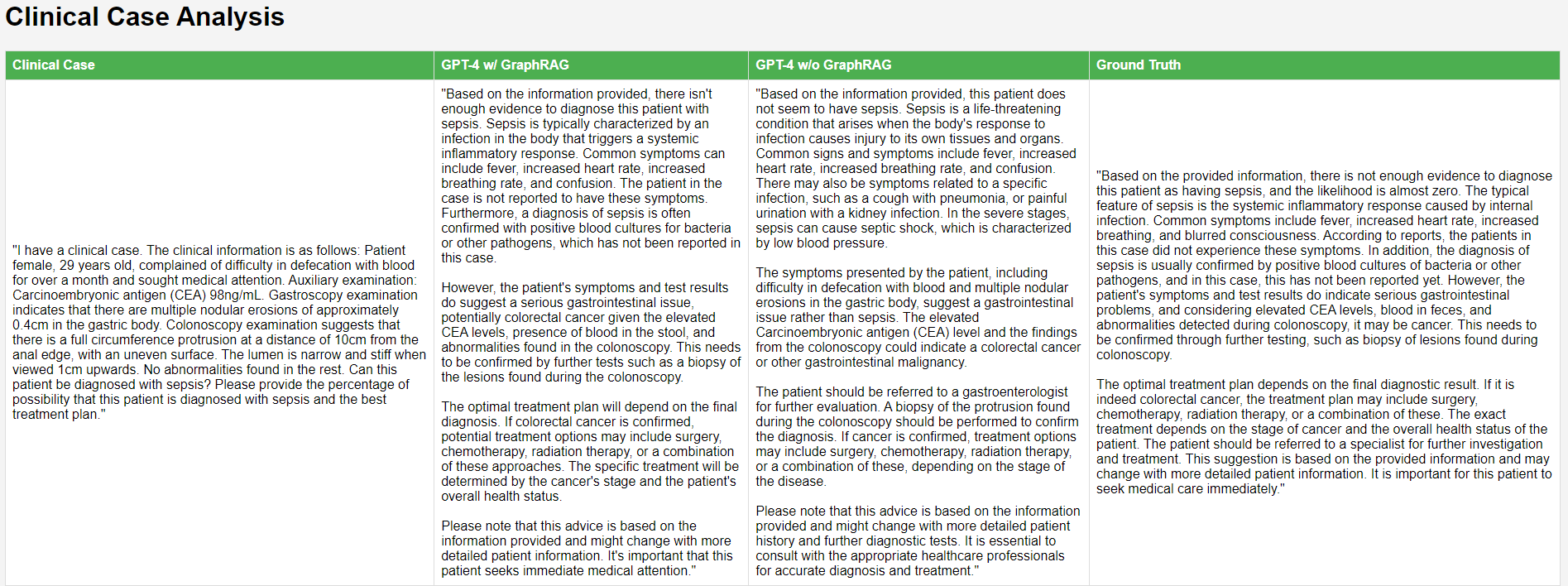


Supplementary Figure S8. The clinical case analysis
